# Interactions in Model Ionic Dyads and Triads Containing Tetrel Atoms

**DOI:** 10.3390/molecules25184197

**Published:** 2020-09-14

**Authors:** Sean A. C. McDowell, Ruijing Wang, Qingzhong Li

**Affiliations:** 1 Department of Biological and Chemical Sciences, Cave Hill Campus, The University of the West Indies, P.O. Box 64, Bridgetown BB11000, Barbados; 2 The Laboratory of Theoretical and Computational Chemistry, School of Chemistry and Chemical Engineering, Yantai University, Yantai 264005, China; wangrj1995@sina.com

**Keywords:** tetrel bond, cooperativity, NBO, EDA

## Abstract

The interactions in model ionic YTX_3_···Z (Y = NC, F, Cl, Br; X = F, Cl, Br, Z = F^−^, Cl^−^, Br^−^, Li^+^) dyads containing the tetrel atoms, T = C, Si, Ge, were studied using ab initio computational methods, including an energy decomposition analysis, which found that the YTX_3_ molecules were stabilized by both anions (via tetrel bonding) and cations (via polarization). For the tetrel-bonded dyads, both the electrostatic and polarization forces make comparable contributions to the binding in the C-containing dyads, whereas, electrostatic forces are by far the largest contributor to the binding in the Si- and Ge-containing analogues. Model metastable Li^+^···NCTCl_3_···F^−^ (T = C, Si, Ge) triads were found to be lower in energy than the combined energy of the Li^+^ + NCTCl_3_ + F^−^ fragments. The pair energies and cooperative energies for these highly polar triads were also computed and discussed.

## 1. Introduction

There is much interest nowadays in noncovalent interactions since they play a major role in many biological and chemical processes and also govern the behaviour of individual molecules in clusters of varying sizes and shapes. The hydrogen bond is the most well known and the most widely-studied noncovalent interaction [1,2,3,4,5,6], but interest has grown considerably in sigma-hole interactions over the last 15 years or so, with much attention initially given to halogen bonding [7,8,9,10,11,12,13,14,15,16].

However, many studies have been reported for a wide range of other sigma–hole interactions besides halogen bonds; the main interest of the present study is that of tetrel bonds, where the positive sigma-hole arises from a Group 14 atom (i.e., C, Si, Ge) covalently-bonded to a more electronegative atom or group of atoms [17,18,19,20,21]. Interest in tetrel bonding has been steadily growing over the last few years, especially since the tetrahedral configuration of the *sp*^3^ hybridized carbon atom is central to a significant portion of organic chemistry. Insights gained from studies of tetrel bonding may find potentially useful applications in fields such as organic synthesis and supramolecular chemistry; for example, tetrel bonding appears to be relevant for the well-known S_N_2 organic reaction [18], hydrophobic interactions [22], and in protein folding and ligand-acceptor interactions [19,23,24].

Cooperativity is an important feature of noncovalent interactions, where two or more separate interactions mutually reinforce each other [25,26]. In some situations, a diminutive effect is achieved, but enhancement of the combined interactions usually occurs. The tetrel bond may be strengthened by cooperative effects in molecular clusters with three or more interacting species [27,28,29,30,31], via hydrogen bonds [32], chalcogen bonds [33], or strong alkaline-earth bonds [34,35], to name a few.

A particular focus of the present study is tetrel-bonded clusters where the positive sigma–hole arising from the tetrel atom binds a model anion. Studies involving interactions between the sigma–hole regions of tetrel atoms and various anions, including halide anions, have been documented before in the literature [18,27,36,37,38,39,40,41]. A previous study of model ionic dyads X···AH_3_-Y (X = F^−^, Cl^−^, Br^−^, Li^+^ and Be^2+^; A = C, Si, Ge; Y = F, Cl, Br) reported on stable anionic and cationic dyads, with the strength of the interaction increasing as a function of the electric field of the ion [42]. A more recent study of model ionic XCCl_3_···Y (X = NC, F, Cl, Br; Y = ion) dyads also found that both anionic and cationic dyads were energetically stable, despite repulsive electrostatic interactions in the latter; an unusual metastable insertion triad, Li^+^···NCCCl_3_···F^−^, was also predicted [43].

In the present work, we extend our investigation to a series of model ionic dyads YTX_3_···Z (Y = NC, F, Cl, Br; T = C, Si, Ge; Z = F^−^, Li^+^), which contain the tetrel atoms C, Si, and Ge, using ab initio computational methods. These dyads allow us to explore the effect on the binding strength and optimized structure of the YTX_3_ molecule as the tetrel atom changes from C to Si to Ge; i.e., from nonmetal to metalloid to semiconductor. We also studied the tetrel-bonded ionic dyads NCCX_3_···Z (X = F, Cl, Br; Z = F^−^, Cl^−^, Br^−^) in order to assess the variation of the interaction strength and structure of the tetrahedral NCCX_3_ molecule when (i) the halogen atoms surrounding the central C atom are changed from F to Cl to Br, and (ii) the anion Z is changed from F^−^ to Cl^−^ to Br^−^.

We then extended the study to neutral, but highly polar, model triads formed by the insertion of NC-TCl_3_ between Li^+^ and F^−^ ions. The Li^+^ atom binds strongly to the N lone pair via electrostatic forces, whereas F^-^ binds to the central T atom via a tetrel bond. We examined the structural changes that occur when the constituent dyads form the triads, as well as the variation in the structure and interaction strength of the central NC-TCl_3_ molecule as the tetrel atom is varied from C to Si to Ge (due to the interaction of the oppositely charged ions). We also assessed the cooperative effects of the noncovalent interactions in the model triad. The computational methodology used in this work is outlined in the following section.

## 2. Discussion

Table 1 shows that, for a fixed tetrel atom, the interaction energy for the anionic Y-TCl_3_···F^−^ dyads decreases going from Y = NC to F (i.e., as the magnitude of the sigma–hole originating from the Y-T bond decreases). However, the interaction then strengthens slightly going down the table from Y = F to Br; this increased binding is probably due to the increasing YTCl_3_ polarizability as Y becomes larger. The T···F^−^ separation is consistent with the trend for *E*^int^; i.e., increase in the T···F^−^ distance from NC to F, and then a decrease from F to Br.

It should be noted that for all Y-TCl_3_···F^−^ dyads, the Y-T bond is elongated, which suggests the displacement of charge into the antibonding σ*(Y-T) orbital. For T = C, the change in the Y-T bond length, ∆*R*(Y-T), increases from NC to F to Cl to Br, and these Y-T bond elongations are correlated with the corresponding T-Cl bond contractions, which increase in magnitude from Y = NC to Br. However, For T = Si and Ge, ∆*R*(Y-T) decreases from NC to F, then increases from F to Cl to Br (which is the same trend for the intermolecular separation). However, for T = Si and Ge, the T-Cl bond is elongated, with the magnitude of elongation decreasing (as the electron-withdrawing ability of Y diminishes from NC to Br); in these dyads the N≡C bond is slightly elongated as charge is shifted towards N≡C by the electric field of the F^−^ anion.

The Si and Ge dyads have much larger interaction energies than the C dyads (by an order of magnitude), with the Si analogues more strongly bound than their Ge counterparts. Consequently, the T···F^−^ distances in the Si and Ge dyads are substantially smaller than their C analogues. Both Si···F and Ge···F distances are close to the sum of their covalent radii, thus the relevant bonds are likely to be covalent bonds.

**Figure 1:**
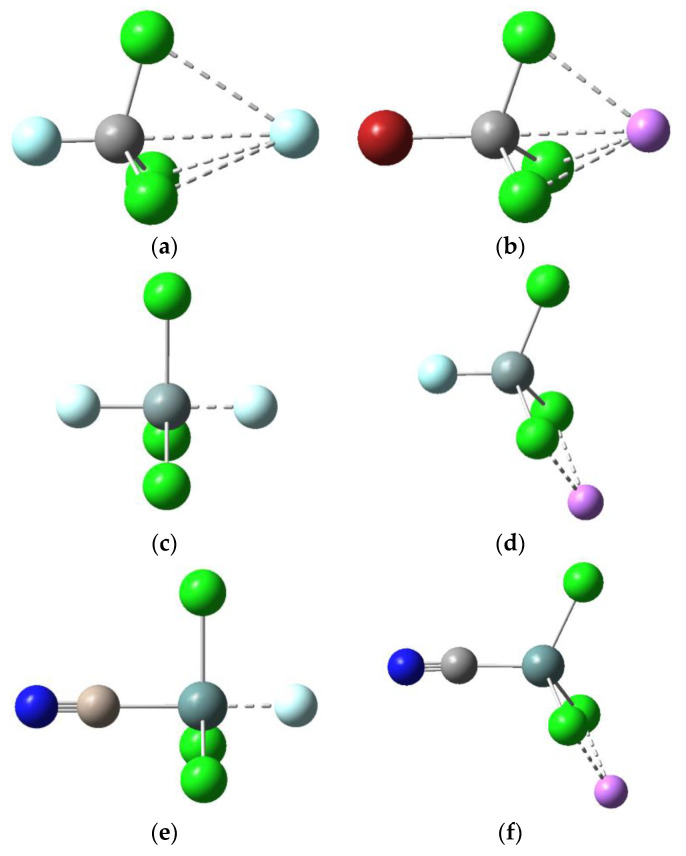
Typical optimized structures for anionic Y-TX_3_···F^−^ and cationic Y-TX_3_···Li^+^ dyads: (**a**) F-CCl_3_···F^−^; (**b**) Br-CCl_3_···Li^+^; (**c**) F-SiCl_3_···F^−^; (**d**) F-SiCl_3_···Li^+^; (**e**) NC-GeCl_3_···F^−^; (**f**) NC-GeCl_3_···Li^+^.

The Y-T-Cl angles in Table 1 and the optimized geometries for the anionic dyads shown in Figure 1 indicate that the Si- and Ge-containing dyads have quite different structures from their C analogues. Figure 1 shows the typical tetrahedral structure for Y-CCl_3_ (<Y-T-Cl = 107°), whereas a trigonal bipyramidal structure for Y-SiCl_3_ and Y-GeCl_3_ is evident, with the Y-T bond perpendicular to one of the T-Cl bonds. All isolated Y-TCl_3_ molecules were optimized to the tetrahedral structures typical of *sp*^3^-hybridized tetrel atoms. However, on complexation the C-containing dyads retain this geometry, whereas the Si- and Ge-containing dyads adopt a more open trigonal bipyramidal structure, which allows the F^−^ anion to approach the tetrel atom more closely in the latter than in the former anionic dyads.

Furthermore, the accessibility of vacant *d* orbitals on Si and Ge allows for strong bonding between these tetrel atoms and the incoming F^−^ anion, leading to increased electron density in the internuclear Si/Ge···F region (as evidenced by the much shorter Si/Ge···F distances relative to C···F, Table 1). For example, NBO analysis (not shown) of NCSiCl_3_ and NCSiCl_3_···F^−^ indicates that Si in the uncomplexed molecule is *sp*^3^-hybridized, whereas in the anionic dyad, substantial *d* orbital participation (by as much as a 15% contribution to the three Si-Cl bonds and 30% contribution to the Si-F bond) suggests *sp*^2^*d* hybridization of the central Si atom.

It is likely that the repulsion between the incoming F^−^ anion and the lone pairs on the Cl atoms surrounding Si causes the Si-Cl bond to elongate and be forced away from F^−^ such that the Y-Si-Cl angle goes from 107° (in the uncomplexed tetrahedral geometry) to 90° (in the bipyramidal structure shown in Figure 1). The YGeCl_3_···F^−^ dyads have a similar structure to the corresponding YSiCl_3_···F^−^ dyads, but smaller interaction energies since F^−^ is about 0.13 Å further away from the tetrel atom T (due to the larger size of Ge and greater repulsion, which limits the interspecies separation).

For the cationic Y-TCl_3_···Li^+^ dyads, the interaction energies span a rather narrow range of 14–25 kcal/mol in magnitude, compared with the more strongly bound anionic dyads, which range between 9 and 129 kcal/mol. The structures for T = C are similar to those of their anionic counterparts (see Figure 1) but the binding in these dyads cannot be attributed to tetrel bonding since the positive sigma-hole and the positive charge on the Li^+^ are opposed (i.e., repulsive). The binding is most likely due to the strong polarization of Y-CCl_3_ by Li^+^ (more about this later). Table 2 shows that *E*^int^ increases as the electron-withdrawing ability of Y decreases going from NC to F to Cl to Br (i.e., as more charge is displaced from Y-CCl_3_ by Li^+^ and the repulsive interaction between the sigma hole and the cation decreases ). Accordingly, the C···Li^+^ separation decreases, the Y-C bond contracts and the C-Cl bond elongates.

For T = Si and Ge, the optimized structures are quite different from their C analogues (Figure 1), with the Y-Si···Li^+^ and Y-Ge···Li^+^ angles being less than 180°, whereas, by contrast, the Y-C···Li^+^ angle is about 180° (as are the Y-T···F^−^ angles in the tetrel-bonded anionic dyads). This optimized geometry allows the Li^+^ to interact favourably with the lone pairs of the two adjacent Cl atoms and at the same time minimizes the repulsion between the cation and the sigma–hole (due to the Y-Si or Y-Ge bonds, both of which would be larger in magnitude than for the Y-C bond). Another interesting observation is that Y-TCl_3_ retains its tetrahedral geometry in the cationic dyads, regardless of the identity of T (the Y-T-X angles range between 111° and 116°, Table 2). By comparison, as noted before, Y-SiCl_3_ and Y-GeCl_3_ are distorted from their tetrahedral geometry in the anionic dyads (where the Y-T-X angles range between 89° and 91°, Table 1) such that trigonal bipyramidal structures are obtained for these dyads.

For the Si- and Ge-containing cationic dyads, *E*^int^ increases from Y = NC to F, then decreases from F to Cl to Br. The T···Li^+^ distances are similar in magnitude (about 3.1–3.2 Å). Similar to the C-containing analogue, the Y-T bond contracts. The two T-Cl bonds closest to Li^+^ are elongated, whereas the more distant T-Cl bond contracts, suggesting charge shift from Y-T and the remote T-Cl towards the T-Cl bonds close to the Li^+^ cation. An elongation of about 0.002 Å for the N≡C bond is obtained for all cationic dyads (i.e., for all T).

The energy decomposition analysis (EDA) results in Table 3 for the anionic Y-TCl_3_···F^−^ dyads show that the electrostatic and polarization terms are the dominant contributors to the binding in the C-containing species, with both terms comparable in magnitude. Both terms are substantially larger in the Si- and Ge-containing dyads, vis-à-vis the C dyad, but the electrostatic contribution is much larger in magnitude than the polarization contribution (generally almost twice as large). Interestingly, the Si-containing species are more strongly bound than their Ge-containing counterparts.

By comparison, the EDA results in Table 4 for the cationic Y-TCl_3_···Li^+^ dyads show that polarization is by far the dominant source of attraction, as suggested in the preceding comments. We note that there is little variation in the value of the polarization contribution in these dyads (ranging in magnitude between 22 and 25 kcal/mol).

A natural bond order (NBO) analysis of the charge distribution for the anionic Y-TCl_3_···F^−^ systems (see Supplementary Materials, Table S1) indicates that charge is mainly transferred from the lone pairs of F^─^ into the antibonding σ* orbital of the Y-T bond. More charge is transferred in the Si and Ge analogues (about 0.26–0.28 *e*) than in the C analogue (about 0.01 *e*), with the magnitude of the charge transfer changing little with varying Y. The sharp contrast between the charge displacement in the C and the charge displacement in the Si/Ge species may be rationalized by comparing the optimized structures in Figure 1. The more open structure for the Si and Ge dyads allows the F^−^ to come much more closely to the tetrel atom of Y-TCl_3_, at the same time minimizing the repulsion between the Cl lone pairs and the incoming F^−^, vis-vis the corresponding C dyads. This is consistent with much shorter Si···F^−^ and Ge···F^−^ distances, compared to the corresponding C···F^−^ distances (see Table 1). The accessibility of the vacant *d* orbitals in Si and Ge for Si···F^−^ and Ge···F^−^ bonding has been mentioned before and is consistent with the large charge transfers in these dyads, which further implies substantial covalent character in the Si···F and Ge···F bonds, compared with the noncovalent C···F bond in the C-containing analogue.

The NBO analysis for the cationic Y-TCl_3_···Li^+^ dyads (see Supplementary Materials, Table S2) suggests that charge is mainly transferred from the Cl lone pair(s) into the vacant 2*s* orbital of Li^+^. More charge is transferred from Y-CCl_3_ (about 0.13–0.14 *e*) compared with Y-SiCl_3_ and Y-GeCl_3_ (about 0.09 *e*). The relative amount of charge transferred in these dyads may be rationalized by considering the Y-TCl_3_···Li^+^ optimized structures in Figure 1 and noting that Li^+^ interacts more closely with all three Cl atoms in the Y-CCl_3_ dyad, whereas the cation only interacts closely with two Cl atoms in the corresponding Si and Ge dyads. A similar amount of charge is transferred from each Cl atom of Y-CCl_3_ to Li^+^ in Y-CCl_3_···Li^+^, whereas significantly more charge is transferred from the two closest Cl atoms of Y-SiCl_3_ and Y-GeCl_3_ to Li^+^ than the more remote Cl atom in the Si and Ge dyads.

The results in Table 5 for the series of model anionic tetrel-bonded NCTX_3_···Z^−^ dyads shows the variation of the tetrel bond strength (and associated structural changes) with changing Z^−^ and X. Not surprisingly, *E*^int^ increases with increasing electric field of the anion Z^−^ (i.e., going from Z^−^ = Br^−^ to Cl^−^ to F^−^) for all NCTX_3_···Z^−^ dyads, with the C···Z^−^ distance decreasing accordingly. Generally, the interspecies interaction increases in the order NCCF_3_ < NCCCl_3_ < NCCBr_3_; i.e., with increasing NCCX_3_ polarizability.

For all dyads, the C-C bond is elongated and the C-X bond compressed, suggesting that charge is transferred into the antibonding σ*(C-C) and out of the antibonding σ*(C-X) orbitals, accompanied by negligible N≡C bond elongations. For fixed NCCX_3_, as the electric field of the anion increases from Br^−^ to Cl^−^ to F^−^, *E*^int^ increases as NCCX_3_ becomes increasingly more polarized, the C-C bond becomes more elongated, the C-X bond more compressed and the C···Z^−^ separation diminishes accordingly.

**Figure 2:**
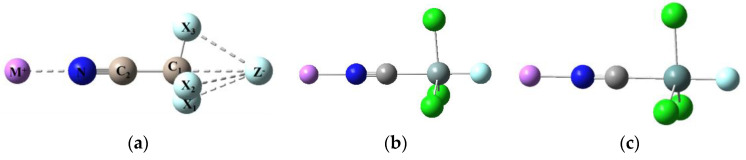
Typical optimized structures for (**a**) M^+^···NC-CX_3_···Z^−^ (M = Li, X= F, Cl, Br; Z = F, Cl, Br), (**b**) Li^+^···NCSiCl_3_···F^−^ and (**c**) Li^+^···NCGeCl_3_···F^−^ triads.

We now turn our attention to the results shown in Table 6 for the model Li^+^···NCTCl_3_···F^−^ triads and their constituent Li^+^···NCTCl_3_ and NCTCl_3_···F^−^ dyad subgroups, which allows us to examine the cooperativity of the two mutual ionic interactions. The optimized stuctures are shown in Figure 2. With reference to the cationic Li^+^···NCTCl_3_ dyad, *E*^int^ is slightly larger in magnitude for the Si and Ge dyads than for the C dyad, the Li^+^···N separation varies little, being about 1.9 Å. The N≡C bond shortens, the C-Cl bond contracts and the C-T bond elongates as charge is withdrawn towards Li^+^; the C-T-Cl angle is largely unaffected relative to the uncomplexed NCTCl_3_ molecule.

With reference to the anionic NCTCl_3_···F^−^ dyad subgroup, Figure 2 shows that the tetrahedral NCCCl_3_ geometry is retained in the triad (∠C-C-Cl = 104°), whereas NCSiCl_3_ and NCGeCl_3_ adopt the trigonal bipyrimidal structures (characteristic of the anionic dyads), with nearly perpendicular C-T and T-Cl bonds. Tetrel bonding appears to strengthen the electrostatic interaction between Li^+^ and the N lone pair on NCTCl_3_, as suggested by the significant decrease in the Li^+^···N separation. Similarly, the tetrel bond between T and F^−^ is enhanced by the Li^+^ interaction as evidenced by the decrease in the T···F^−^ separation.

We note an increase in the C-Cl contraction in Li^+^···NCTCl_3_···F^−^, decreases in the Si-Cl and Ge-Cl extensions in their respective triads, and negligible N≡C bond changes throughout, relative to the anionic dyads. The enhanced C-T bond extensions for all three triads in Table 6 suggest a mutual (positive) cooperative effect. The C-T-Cl angles decrease slightly in going from the anionic dyads to the triad, but the dyad structure of NCTCl_3_ is retained in the triad.

The results in Table 7 for the energetic partitioning of the interaction energy of Li^+^···NCTCl_3_···F^−^ into its constituent pair energies and an estimated cooperative energy are insightful and consistent with the structural changes evident in Table 6. For Li^+^···NCTCl_3_···F^−^, the binding is dominated by the interaction between the Li^+^ and F^−^ ions, with the cooperative energy *E*^coop^ contributing about 12% to the total interaction energy. In fact, the cooperative energies are fairly similar in magnitude (14–17 kcal/mol), but for the Si- and Ge-containing triads, *E*^coop^ only contributes 7% to the total interaction energy.

For Li^+^···NCSiCl_3_···F^−^ and Li^+^···NCGeCl_3_···F^−^, the Li^+^···F^−^ pair energies (*E*_ac_) are similar in magnitude to the pair energy for Li^+^···NCCCl_3_···F^−^, as would be expected. On the other hand, the Li^+^···NCTCl_3_ pair energy (*E*_ab_) for the Si- and Ge-containing triads have the same values and are larger than the corresponding pair energy for the C-containing analogue. Interestingly, *E*_ab_ and *E*_ac_ have almost the same values for Si and Ge.

However, for the Si- and Ge-containing triads, the NCTCl_3_···F^−^ pair interaction (*E*_bc_) is by far the largest contributor to the binding which, as was noted from the EDA results in Table 3, are dominated by electrostatic forces; more than 50% of the binding is due to this very strong interaction, which eclipses the other interactions. This finding further supports the notion that the Si···F and Ge···F bonds in the anionic dyad fragment are mainly covalent in character, and this covalency is enhanced by the binding of Li^+^ to the lone pair of the N atom of NCTCl_3_ in the triads.

It should be noted that the M^+^···YTCl_3_···Z^−^ (M = metal, Z = halogen) triads are likely to be highly metastable. For example, two minima on the potential energy surface of the Li^+^···NCCBr_3_···Br^−^ triad,

NCCBr_3_···BrLi and BrLi···NCCBr_3_, were both found to be lower in energy than the former by 836 and 1089 kcal/mol, respectively. Nonetheless, it may, in principle, be possible to synthesize triads such as these by first making the strongly bound anionic tetrel-bonded YTCl_3_···Z^−^ dyad fragment and then carefully binding the cation M^+^ to the Y group opposite the anion Z^−^.

In conclusion, a series of stable model anionic and cationic dyads YTX_3_···Z (Y = NC, F, Cl, Br; X = F, Cl, Br; Z = F^−^, Li^+^) containing the tetrel atoms, T = C, Si, Ge, were predicted to be energetically stable. Energy decomposition analysis showed that the YTX_3_ molecules were stabilized by tetrel bonding in the anionic dyads and by polarization in the cationic dyads. In the anionic tetrel-bonded YCCl_3_ dyads, both the electrostatic and polarization forces make comparable contributions to the binding, whereas electrostatic forces dominate in the YTCl_3_ (T = Si, Ge) dyads. For the latter dyads, the Si···F and Ge···F bonds appear to have strong covalent character, which is further enhanced in the triads formed when Li^+^ binds to the N lone pair in model Li^+^···NCTCl_3_···F^−^ (T = Si, Ge) triads. The less strongly bound C-containing triads also show cooperative effects but the C···F bond appears to retain its essentially noncovalent character in this triad.

## 3. Computational Methodology

All ab initio calculations were performed at the MP2/aug-cc-pVTZ level of theory using the Gaussian 09 suite of program [44]. Vibrational frequency calculations were performed at the same computational level to confirm that the optimized structures correspond to energetic minima (no imaginary frequencies were found). Interaction energies were calculated using the supermolecular approach and corrected for the basis set superposition error (BSSE) using the counterpoise method proposed by Boys and Bernardi [45]. For dyads, the interaction energy *E*^int^ was calculated using Equation (1) below:

Δ*E*^int^ = *E*_A-B_ − *E*_SP(A)_ − *E*_SP(B)_ (1)

where *E*_A-B_ is the energy of dyad A-B, *E*_SP(A)_ and *E*_SP(B)_ are the single-point energies of monomers A and B obtained from the dyads. For triads, when two interactions coexist, the cooperative energy was calculated using Equation (2).

*E*^coop^ = Δ*E*_A-B-C_ − Δ*E*_A-B_ − Δ*E*_B-C_ − Δ*E*_A-C_ (2)

where Δ*E*_A-B-C_ is the total interaction energy of the triad, Δ*E*_A-B_ and Δ*E*_B-C_ are the interaction energies of the optimized dyads, and Δ*E*_A-C_ is the interaction energy between the two non-bonded molecules A and C in the triad. The orbital interaction and charge transfer between the electron donor and acceptor in the dyads were studied by using the natural bond orbital (NBO) method [46]. The energy decomposition analysis for the dyads was performed by the GAMESS program [47] using the localized molecular orbital-energy decomposition analysis (LMO-EDA) method [48] at the MP2/aug-cc-pVTZ level, with the interaction energy being partitioned into five energy components (electrostatic, exchange, repulsion, polarization, and dispersion).

## Figures and Tables

**Table 1:** MP2/aug-cc-pVTZ parameters for model anionic Y-TCl_3_···F^−^ (Y = NC, F, Cl, Br; T = C, Si, Ge) dyads. The properties with respect to the isolated molecules are the counterpoise-corrected interaction energy (*E*^int^ in kcal/mol), intermolecular separation (*R*(T···F^−^ in Å) and the bond length changes *(*∆*R* in Å). All three T-Cl bond length changes have the same value. The Y-T-Cl angle is also shown.

Dyads	*E* ^int^	*R* (T···F^−^)	Δ*R* (Y-T)	Δ*R* (T-Cl)	Δ*R* (N≡C)	∠Y-T-Cl
NCCCl_3_···F^−^	−14.2	2.826	0.010	−0.007	0.000	107.0
FCCl_3_···F^−^	−9.3	2.894	0.029	−0.010	--	106.9
ClCCl_3_···F^−^	−9.1	2.909	0.050	−0.014	--	107.5
BrCCl_3_···F^−^	−9.5	2.888	0.055	−0.015	--	107.3
NCSiCl_3_···F^−^	−128.6	1.652	0.090	0.102	0.002	89.2
FSiCl_3_···F^−^	−118.6	1.656	0.069	0.110	--	90.0
ClSiCl_3_···F^−^	−121.2	1.658	0.182	0.090	--	90.0
BrSiCl_3_···F^−^	−124.1	1.656	0.228	0.082	--	90.4
NCGeCl_3_···F^−^	−111.8	1.778	0.096	0.090	0.002	90.2
FGeCl_3_···F^−^	−105.9	1.782	0.073	0.088	--	90.0
ClGeCl_3_···F^−^	−107.0	1.782	0.169	0.077	--	90.7
BrGeCl_3_···F^−^	−108.7	1.779	0.201	0.073	--	90.6

“--” indicate that no data is supplied for these values.

**Table 2:** MP2/aug-cc-pVTZ parameters for model cationic Y-TCl_3_···Li^+^ (Y = NC, F, Cl, Br; T = C, Si, Ge) dyads. The properties with respect to the isolated molecules are the counterpoise-corrected interaction energy (*E*^int^ in kcal/mol), intermolecular separation (*R* (T···Li^+^) in Å) and the bond length changes (∆*R* in Å). The Y-T-Cl angle is also shown.

Dyads	*E* ^int^	*R* (T···Li^+^)	Δ*R* (Y-T)	Δ*R* (T-Cl) ^1^	Δ*R* (T-Cl) ^2^	Δ*R* (N≡C)	∠Y-T-Cl
NCCCl_3_···Li^+^	−14.3	2.664	−0.013	0.015	0.015	0.002	110.9
FCCl_3_···Li^+^	−17.3	2.637	−0.031	0.017	0.017	--	110.4
ClCCl_3_···Li^+^	−21.0	2.637	−0.044	0.022	0.022	--	111.7
BrCCl_3_···Li^+^	−21.8	2.630	−0.044	0.022	0.022	--	111.8
NCSiCl_3_···Li^+^	−21.4	3.138	−0.026	−0.037	0.050	0.002	114.1
FSiCl_3_···Li^+^	−24.2	3.119	−0.018	−0.040	0.046	--	113.8
ClSiCl_3_···Li^+^	−22.3	3.122	−0.036	−0.036	0.053	--	114.9
BrSiCl_3_···Li^+^	−18.6	3.120	−0.041	−0.034	0.056	--	115.2
NCGeCl_3_···Li^+^	−21.7	3.171	−0.026	−0.036	0.052	0.002	115.5
FGeCl_3_···Li^+^	−25.4	3.154	−0.019	−0.038	0.048	--	114.0
ClGeCl_3_···Li^+^	−24.3	3.157	−0.035	−0.035	0.055	--	115.9
BrGeCl_3_···Li^+^	−20.7	3.155	−0.089	−0.033	0.058	--	116.4

^1^ The change of the T-Cl bond furthest away from the Li^+^ atom. ^2^ The changes of the two T-Cl bonds closest to the Li^+^ atom. “--” indicate that no data is supplied for these values.

**Table 3:** MP2/aug-cc-pVTZ energy decomposition analysis (EDA) for model anionic Y-TX_3_···F^−^ (Y = NC, F, Cl, Br; T = C, Si, Ge; X = Cl) dyads.

Dyads	*E* ^elec^	*E* ^ex^	*E* ^rep^	*E* ^pol^	*E* ^disp^
NCCCl_3_···F^−^	−19.7	−35.2	59.4	−16.5	−2.3
FCCl_3_···F^−^	−13.2	−30.4	50.8	−14.2	−2.4
ClCCl_3_···F^−^	−11.7	−30.8	51.4	−15.1	−3.0
BrCCl_3_···F^−^	−12.0	−31.8	53.1	−15.8	−3.1
NCSiCl_3_···F^−^	−207.7	−194.6	394.4	−130.6	8.5
FSiCl_3_···F^−^	−199.1	−195.9	395.9	−128.2	7.5
ClSiCl_3_···F^−^	−201.1	−196.0	396.2	−128.8	7.4
BrSiCl_3_···F^−^	−203.2	−195.2	395.0	−129.6	7.8
NCGeCl_3_···F^−^	−191.7	−170.8	350.0	−106.5	7.3
FGeCl_3_···F^−^	−184.9	−167.7	343.4	−103.2	6.8
ClGeCl_3_···F^−^	−186.2	−170.1	347.8	−104.6	6.4
BrGeCl_3_···F^−^	−188.2	−170.9	349.8	−105.8	6.6

**Table 4:** MP2/aug-cc-pVTZ energy decomposition analysis (EDA) for model cationic Y-TX_3_···Li^+^ (Y = NC, F, Cl, Br; T = C, Si, Ge; X = Cl) dyads.

Dyads	*E* ^elec^	*E* ^ex^	*E* ^rep^	*E* ^pol^	*E* ^disp^
NCCCl_3_···Li^+^	6.4	−2.4	7.4	−23.1	−3.1
FCCl_3_···Li^+^	1.9	−2.5	7.5	−22.3	−2.6
ClCCl_3_···Li^+^	−0.9	−2.9	8.7	−24.3	−2.2
BrCCl_3_···Li^+^	−1.3	−3.0	9.0	−25.1	−2.2
NCSiCl_3_···Li^+^	−1.4	−3.6	11.0	−23.9	−1.2
FSiCl_3_···Li^+^	−5.4	−3.8	11.5	−23.2	−1.1
ClSiCl_3_···Li^+^	−7.6	−4.1	12.5	−24.7	−1.0
BrSiCl_3_···Li^+^	−1.3	−2.6	7.8	−24.6	−2.4
NCGeCl_3_···Li^+^	−3.3	−4.0	12.0	−24.7	−1.2
FGeCl_3_···Li^+^	−5.3	−3.9	11.8	−23.7	−1.2
ClGeCl_3_···Li^+^	−8.5	−4.4	13.1	−25.4	−0.9
BrGeCl_3_···Li^+^	−2.6	−2.7	8.0	−25.3	−2.3

**Table 5:** MP2/aug-cc-pVTZ parameters for model anionic NC-CX_3_···Z^−^ (X = Br, Cl, F; Z = Br, Cl, F) dyads. The properties with respect to the isolated molecules are the counterpoise-corrected interaction energy (*E*^int^ in kcal/mol), intermolecular separation (*R* in Å) and the bond length changes (∆*R* in Å). All three C-X bond length changes have the same value.

Dyads	*E* ^int^	*R* (C···Z^−^)	∆*R* (C-C)	∆*R* (C-X)	∆*R* (N≡C)
NCCBr_3_···Br^−^	−9.5	3.756	0.003	−0.004	0.0002
NCCBr_3_···Cl^−^	−10.7	3.560	0.004	−0.005	0.0002
NCCBr_3_···F^−^	−17.0	2.762	0.008	−0.008	0.0004
NCCCl_3_···Br^−^	−8.1	3.809	0.005	−0.004	0.0000
NCCCl_3_···Cl^−^	−9.0	3.619	0.006	−0.004	0.0000
NCCCl_3_···F^−^	−14.2	2.826	0.010	−0.007	0.0002
NCCF_3_···Br^−^	−5.4	3.732	0.017	−0.006	0.003
NCCF_3_···Cl^−^	−6.2	3.554	0.019	−0.007	0.001
NCCF_3_···F^−^	−10.4	2.834	0.028	−0.009	0.000

**Table 6:** MP2/aug-cc-pVTZ parameters for model Li^+^···NC-TCl_3_···F^−^ triads, Li^+^···NC-TCl_3_ and NC-TCl_3_···F^−^ dyads (T = C, Si, Ge). The bond length changes in the triads (Δ*R*) are computed relative to the bond length in the isolated optimized NC-TCl_3_ molecule.

	*E* ^int^	*R* (Li^+^···N)	*R* (T···F^−^)	*R* (Cl···F^−^)	Δ*R* (T-Cl)	Δ*R* (C-T)	Δ*R* (N-C)	∠C-T-Cl
Triads								
Li^+^···NCCCl_3_···F^−^	−112.5	1.858	2.475	2.673	−0.014	0.017	−0.005	103.7
Li^+^···NCSiCl_3_···F^−^	−254.4	1.830	1.631	2.742	0.074	0.167	−0.003	86.2
Li^+^···NCGeCl_3_···F^−^	−236.3	1.834	1.755	2.866	0.059	0.206	−0.002	86.6
Dyads								
Li^+^···NCCCl_3_	−33.9	1.943	--	--	−0.009	0.011	−0.007	107.5
Li^+^···NCSiCl_3_	−38.1	1.936	--	--	−0.017	0.059	−0.007	105.2
Li^+^···NCGeCl_3_	−37.3	1.939	--	--	−0.016	0.063	−0.007	105.3
NCCCl_3_···F^−^	−14.2	--	2.826	2.860	−0.007	0.010	0.000	107.0
NCSiCl_3_···F^−^	−128.6	--	1.652	2.709	0.102	0.090	0.002	89.2
NCGeCl_3_···F^−^	−111.8	--	1.778	2.819	0.090	0.096	0.002	90.2

“--” indicate that no data is supplied for these values.

**Table 7:** MP2/aug-cc-pVTZ total interaction energy (*E*^total^), pair interaction energies (*E*_ab_, *E*_bc,_ *E*_ac_) and cooperative energies (*E*^coop^) for the model Li^+^···NC-TCl_3_···F^−^ (T = C, Si, Ge) ternary systems. All energies are in kcal/mol. *E*^total^ = *E* − (*E*_a_ + *E*_b_ + *E*_c_) and *E*^coop^ = *E*^total^ − (*E*_ab_ + *E*_bc_ + *E*_ac_), where *E* is the total energy of the triad (a···b···c), *E*_a_ is the energy of species a and *E*_ab_ is the interaction energy of the dyad a···b. All energies are computed for the geometries that each species adopts in the optimized triad. The percentage contribution of *E*^coop^ to the total interaction energy is given in brackets.

Triads (a···b···c)	*E* ^total^	*E*_ab_	*E*_bc_	*E*_ac_	*E* ^coop^
Li^+^···NCCCl_3_···F^−^	−112.5	−35.7	−15.0	−47.8	−14.0 (12%)
Li^+^···NCSiCl_3_···F^−^	−254.4	−49.3	−137.8	−50.2	−17.1 (7%)
Li^+^···NCGeCl_3_···F^−^	−236.3	−49.4	−121.6	−48.6	−16.7 (7%)

## Supplementary Materials

The following are available online, Table S1: NBO charge transfer (CT in e) and second-order perturbation theory stabilization energy (E(2) in kcal/mol) for selected orbital transitions in the anionic Y-TCl_3_···F^−^ dyads, Table S2: NBO charge transfer (CT in e) and second-order perturbation theory stabilization energy (E(2) in kcal/mol) for selected orbital transitions in the cationic Y-TCl_3_···Li+ dyads.
