# Supplementary material for: Interactions in Model Ionic Dyads and Triads Containing Tetrel Atoms
Source: Molecules. 2020 Sep 14;25(18):4197. doi: 10.3390/molecules25184197 (PMC7570900; doi:10.3390/molecules25184197)
Supplement: Supplementary file 1 [file molecules-25-04197-s001.pdf]

Supporting Information

Table S1: NBO charge transfer (CT in *e*) and second-order perturbation theory stabilization energy ($E^{(2)}$ in kcal/mol) for selected orbital transitions in the anionic Y-TCl_3…F^- dyads.

dyads	CT	Orbital transition	$E^{(2)}$
$NCCCl_3 \cdots F^-$	0.0119	$LP_{F^-} \rightarrow BD^*_{C\text{-}C}$	3.13
$FCCl_3 \cdots F^-$	0.0098	$LP_{F^-} \rightarrow BD^*_{C\text{-}F}$	2.27
$ClCCl_3 \cdots F^-$	0.0112	$LP_{F^-} \rightarrow BD^*_{C\text{-}Cl}$	2.80
$BrCCl_3 \cdots F^-$	0.0123	$LP_{F^-} \rightarrow BD^*_{C\text{-}Br}$	3.32
$NCSiCl_3 \cdots F^-$	0.2792	$LP_{F^-} \rightarrow BD^*_{Si\text{-}C}$	12.56
$FSiCl_3 \cdots F^-$	0.2679	$LP_{F^-} \rightarrow BD^*_{Si\text{-}F}$	11.15
$ClSiCl_3 \cdots F^-$	0.2663	$LP_{F^-} \rightarrow BD^*_{Si\text{-}Cl}$	10.82
$BrSiCl_3 \cdots F^-$	0.2648	$LP_{F^-} \rightarrow BD^*_{Si\text{-}Br}$	10.39
$NCGeCl_3 \cdots F^-$	0.2763	$LP_{F^-} \rightarrow BD^*_{Ge\text{-}C}$	13.43
$FGeCl_3 \cdots F^-$	0.2663	$LP_{F^-} \rightarrow BD^*_{Ge\text{-}F}$	9.91
$ClGeCl_3 \cdots F^-$	0.2653	$LP_{F^-} \rightarrow BD^*_{Ge\text{-}Cl}$	9.51
$BrGeCl_3 \cdots F^-$	0.2639	$LP_{F^-} \rightarrow BD^*_{Ge\text{-}Br}$	9.38

Table S2: NBO charge transfer (CT in *e*) and second-order perturbation theory stabilization energy ($E^{(2)}$ in kcal/mol) for selected orbital transitions in the cationic Y-TCl$_3$…Li$^+$ dyads.

dyads	CT		$E^{(2)}$		$E^{(2)}$		$E^{(2)}$
NCCCl$_3$···Li$^+$	0.1321	$LP_{Cl1}\rightarrow LP^*_{Li^+}$	36.62	$LP_{Cl2}\rightarrow LP^*_{Li^+}$	36.98	$LP_{Cl3}\rightarrow LP^*_{Li^+}$	37.01
FCCl$_3$···Li$^+$	0.1289	$LP_{Cl1}\rightarrow LP^*_{Li^+}$	37.90	$LP_{Cl2}\rightarrow LP^*_{Li^+}$	37.93	$LP_{Cl3}\rightarrow LP^*_{Li^+}$	37.95
ClCCl$_3$···Li$^+$	0.1411	$LP_{Cl1}\rightarrow LP^*_{Li^+}$	40.66	$LP_{Cl2}\rightarrow LP^*_{Li^+}$	41.08	$LP_{Cl3}\rightarrow LP^*_{Li^+}$	41.14
BrCCl$_3$···Li$^+$	0.1454	$LP_{Cl1}\rightarrow LP^*_{Li^+}$	41.41	$LP_{Cl2}\rightarrow LP^*_{Li^+}$	41.41	$LP_{Cl3}\rightarrow LP^*_{Li^+}$	41.41
NCSiCl$_3$···Li$^+$	0.0871	$LP_{Cl1}\rightarrow LP^*_{Li^+}$	33.87	$LP_{Cl2}\rightarrow LP^*_{Li^+}$	33.23	$LP_{Cl3}\rightarrow LP^*_{Li^+}$	--
FSiCl$_3$···Li$^+$	0.0884	$LP_{Cl1}\rightarrow LP^*_{Li^+}$	35.85	$LP_{Cl2}\rightarrow LP^*_{Li^+}$	35.71	$LP_{Cl3}\rightarrow LP^*_{Li^+}$	--
ClSiCl$_3$···Li$^+$	0.0923	$LP_{Cl1}\rightarrow LP^*_{Li^+}$	37.16	$LP_{Cl2}\rightarrow LP^*_{Li^+}$	37.15	$LP_{Cl3}\rightarrow LP^*_{Li^+}$	0.08
BrSiCl$_3$···Li$^+$	0.0939	$LP_{Cl1}\rightarrow LP^*_{Li^+}$	37.68	$LP_{Cl2}\rightarrow LP^*_{Li^+}$	37.68	$LP_{Cl3}\rightarrow LP^*_{Li^+}$	0.09
NCGeCl$_3$···Li$^+$	0.0918	$LP_{Cl1}\rightarrow LP^*_{Li^+}$	36.59	$LP_{Cl2}\rightarrow LP^*_{Li^+}$	36.64	$LP_{Cl3}\rightarrow LP^*_{Li^+}$	0.05
FGeCl$_3$···Li$^+$	0.0905	$LP_{Cl1}\rightarrow LP^*_{Li^+}$	36.88	$LP_{Cl2}\rightarrow LP^*_{Li^+}$	37.14	$LP_{Cl3}\rightarrow LP^*_{Li^+}$	--
ClGeCl$_3$···Li$^+$	0.0960	$LP_{Cl1}\rightarrow LP^*_{Li^+}$	39.10	$LP_{Cl2}\rightarrow LP^*_{Li^+}$	39.15	$LP_{Cl3}\rightarrow LP^*_{Li^+}$	--
BrGeCl$_3$···Li$^+$	0.0896	$LP_{Cl1}\rightarrow LP^*_{Li^+}$	35.82	$LP_{Cl2}\rightarrow LP^*_{Li^+}$	35.83	$LP_{Cl3}\rightarrow LP^*_{Li^+}$	0.07
